# Respondent Demographics and Contraceptive Use Patterns in the United States: A National Survey of Family Growth Analysis

**DOI:** 10.7759/cureus.53121

**Published:** 2024-01-28

**Authors:** Barbara Prol, Sarah Klein, Christopher Rennie, Sanela Andelija

**Affiliations:** 1 Medicine, Nova Southeastern University Dr. Kiran C. Patel College of Osteopathic Medicine, Clearwater, USA; 2 Department of Obstetrics and Gynecology, HCA Florida Brandon Hospital, Brandon, USA

**Keywords:** social determinants of health, family planning, sexually transmitted infections, unintended pregnancy, contraception

## Abstract

Introduction: Contraception is an important tool for helping to prevent both unintended pregnancies and sexually transmitted infections (STIs). Medical costs related to STIs are high and impose a large burden on both patients and the healthcare system. In addition, unintended pregnancies account for a large portion of pregnancies in the United States (US) and are associated with adverse maternal and infant health outcomes. Both STIs and unintended pregnancies are continuous public health concerns, and this study aims to identify patterns in contraceptive method use in relation to specific social determinants of health.

Methods: Utilizing the Centers for Disease Control and Prevention (CDC)’s 2017-2019 National Survey of Family Growth report on current contraceptive status, we isolated data from 3,572 respondents who reported using one of the following contraceptive methods: oral contraceptive pills (OCPs), male condoms, partner’s vasectomy, female sterilization, withdrawal, medroxyprogesterone acetate injections (Depo-Provera), hormonal implant, or an intrauterine device (IUD). We analyzed these contraceptive methods among age, race, education, marital status, and insurance status. Data were analyzed in RStudio 2022.02.0 (RStudio Team, RStudio: Integrated Development for R. RStudio, PBC, Boston, MA) through a test of equal proportions for a significance of *P *< 0.05. A concurrent Yates' continuity correction was performed in order to limit erroneous significant findings based on small sample sizes where applicable. The study conception and data analysis were performed independently with oversight from our preceptor at HCA Florida Brandon Hospital, Brandon, Florida.

Results: There were statistically significant differences for all our selected methods of contraception across different age groups. There were statistically significant differences for OCPs, male condoms, partner’s vasectomy, female sterilization, Depo-Provera, hormonal implant, and IUD across different race groups and different insurance statuses. There were statistically significant differences for OCPs, male condoms, partner’s vasectomy, female sterilization, withdrawal, hormonal implant, and IUD across different education levels and different marital statuses.

Conclusion: This analysis highlights gaps that are present in female reproductive autonomy through the statistical differences in contraceptive methods across various demographics and warrants continued focus on the role that social determinants of health play in the prevention of unintended pregnancies and STIs. In order to promote fairness and equality in healthcare, it is essential to increase education on these topics both within and beyond medical settings. This effort aims to provide patients with equitable access to healthcare and attempt to address health disparities that are prevalent in multiple different sectors.

## Introduction

Contraception is an important tool for helping to prevent both unintended pregnancies and sexually transmitted infections (STIs) both across the world and in the United States (US). Unintended pregnancies account for nearly 50% of all US pregnancies and are associated with both maternal and infant adverse health outcomes [1]. It is important to note that an unintended pregnancy does not always mean undesired. However, whether or not the unintended pregnancy is undesired, the consequences of an unintended pregnancy are broad and serious. An unintended pregnancy can lead to difficult situations and decisions, including abortion, adoption, or undergoing financial, emotional, and physical tolls [2]. Apart from these consequences, the health risks associated with such pregnancies can be serious and fatal.

Maternal mortality is an increasing issue in the US. The maternal mortality rate in 2021 was 32.9 deaths per 100,000 live births, compared with 23.8 in 2020 and 20.1 in 2019 [3]. According to the Centers for Disease Control and Prevention (CDC), while increases in pregnancy-related mortality may have been affected by changes in computerized data, coding, and the addition of the pregnancy checkbox to death records, the most common causes of pregnancy-related mortality include infection or sepsis, cardiomyopathy, hemorrhage, thrombotic pulmonary or other embolism, hypertensive disorders of pregnancy, amniotic fluid embolism, cerebrovascular accidents, and “other cardiovascular conditions” [4]. Increased cesarean delivery rates, systemic racism, and reduced access to abortion services may also contribute to maternal mortality and should be considered [5].

In 2018, the prevalence of STIs in the US was estimated to be 67.6 million, which accounts for approximately 20% of the US population [6]. Incident STIs in 2018 imposed an estimated $15.9 billion burden in lifetime medical costs [7]. Between 2017 and 2021, the prevalence of gonorrhea has increased by 28%, total syphilis has increased by 74%, and congenital syphilis has increased to more than 203% [8]. While this may be a reflection of increased diagnosis and reporting, the burden to the patients and the healthcare system is the same. STIs can lead to serious complications, including pelvic inflammatory disease, infertility, congenital infections such as syphilis or newborn gonococcal conjunctivitis, ectopic pregnancies, and infant death [8,9].

Since both STIs and unintended pregnancies are largely preventable with proper use of contraceptive devices, education surrounding the use and benefits of contraception is needed, and cost-effective contraceptive methods are imperative. This study aims to identify disparities in contraceptive usage based on demographic factors, such as age, race, education, marital status, and insurance status. The goal is to increase awareness and recognition of these factors in contraceptive decision-making.

This article was previously presented as a poster and meeting abstract at the American Congress of Obstetrics and Gynecology 2023 District XII Annual Meeting on August 11, 2023.

## Materials and methods

This study utilized retrospective female respondent data from 6,141 women aged 15-49 from the CDC’s 2017-2019 National Survey of Family Growth (NSFG) report on current contraceptive status [10]. User agreements in accordance with the CDC were completed, and permission was obtained for the use of this dataset. In utilizing this data, it is important to note that “female respondent data” and any use of the term “women," for the purposes of this analysis, refers to individuals who classified themselves as the female sex when responding to this survey but that this term may include transgender men and all others seeking gynecologic care. The study conception and data analysis were performed independently with oversight from our preceptor at HCA Florida Brandon Hospital, Brandon, Florida.

Out of the total number of respondents, data were isolated from 3,572 respondents who reported using one of the following contraceptive methods: oral contraceptive pills (OCPs), male condoms, partner’s vasectomy, female sterilization, withdrawal, medroxyprogesterone acetate (Depo-Provera) injections, hormonal implant, or an intrauterine device (IUD). Contraceptive methods used in the last 12 months were analyzed among age, race, education, marital status, and insurance status. It is important to note that during the survey administration, one woman was able to mark multiple forms of contraception where applicable. Age was divided into four groups: 15-19, 20-29, 30-39, and 40-49. Race was divided into four groups: White, Black or African American, Hispanic, or other race groups. Education was categorized into eight groups: high school level, high school degree, college level, undergraduate, and higher education degrees. Master’s, doctorate, and professional degrees were grouped under higher education degrees for analysis purposes. Marital status was divided into five groups: married to a man, not married but living with a man, divorced, separated, or never been married. Widowed women were excluded from this study because the sample size was too small. Insurance status was divided into four groups as listed in the NSFG: private/Med-Gap, Medicaid/Children's Health Insurance Program (CHIP)/state, Medicare/military/government, and single service/Indian/uninsured.

Data were analyzed in RStudio 2022.02.0 (RStudio Team, RStudio: Integrated Development for R. RStudio, PBC, Boston, MA) through a test of equal proportions for a significance of P < 0.05. A concurrent Yates' continuity correction was performed in order to limit erroneous significant findings based on small sample sizes where applicable.

## Results

**Figure 1:**
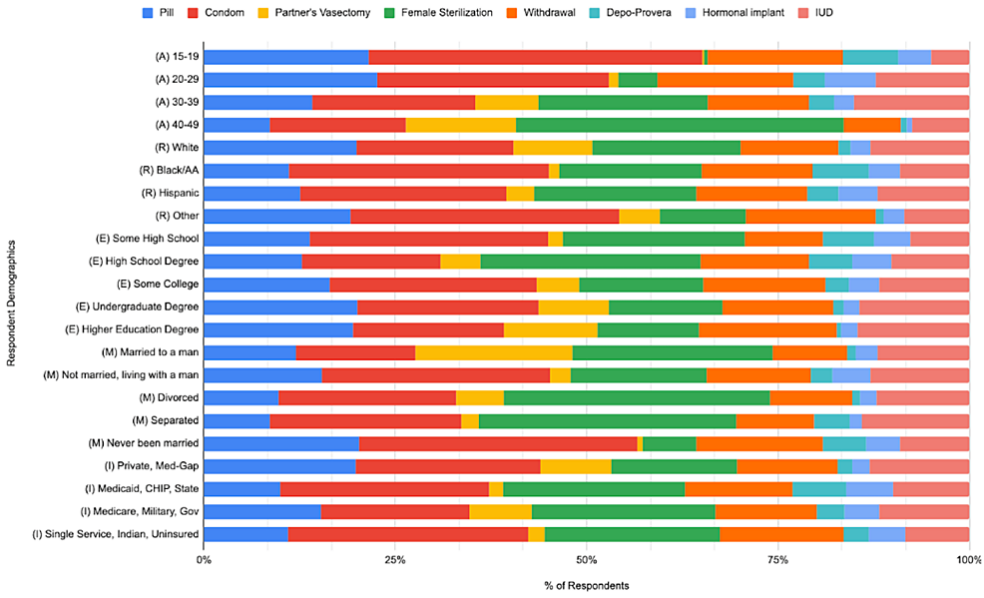
Patterns of contraceptive use across age, race, education, marital status, and insurance status

Out of 6,141 total female respondents, 3,572 (58.17%) respondents reported using OCPs, male condoms, partner’s vasectomy, sterilization, withdrawal, Depo-Provera, hormonal implant, and/or IUD within the last year. P-values for all categories are found in Table 1. Raw percentage results for respondent data across these options and demographics are found in Table 2. A graphical representation of these percentage results and patterns of contraceptive choice is viewed in Figure 1.

**Table 1:** Test of equal proportions across various social determinants of health and contraceptive options *Indicates a significant P value < 0.05.  †Indicates where the sample size was too small to calculate an accurate P value. IUD: intrauterine device; OCP: oral contraceptive pill; SDOH: social determinant of health

SDOH	OCP	Male condoms	Partner’s vasectomy	Female sterilization	Withdrawal	Depo-Provera	Hormonal implant	IUD
Age	<0.001*	<0.001*	<0.001*	<0.001*	<0.001*	<0.001*	<0.001*	<0.001*
Race	<0.001*	<0.001*	<0.001*	0.005*	0.51	<0.001*	0.008*	0.005*
Education	<0.001*	<0.001*	<0.001*	<0.001*	0.001*	-^†^	0.002*	<0.001*
Marital status	<0.001*	<0.001*	<0.001*	<0.001*	<0.001*	-	0.002*	0.02*
Insurance	<0.001*	0.002*	<0.001*	<0.001*	.47	<0.001*	<0.001*	0.002*

**Table 2:** Raw percentage results from respondent data regarding contraceptive options across different demographics IUD: intrauterine device; SDOH: social determinant of health; CHIP: Children's Health Insurance Program *It is important to note that during survey administration, one woman could mark multiple forms of contraception where applicable. In this way, frequency sums may exceed 100% as all percentages were created from the total number of respondents in the category as opposed to the total number of responses.

SDOH	SDOH categories	Pill	Condom	Partner's vasectomy	Female sterilization	Withdrawal	Depo-Provera	Hormonal implant	IUD
Age	15-19 (N=334)	28.44% (95)	57.49% (192)	0.30% (1)	0.60% (2)	23.35% (78)	9.58% (32)	5.69% (19)	6.59% (22)
	20-29 (N=1139)	25.90% (295)	34.50% (393)	1.49% (17)	5.79% (66)	20.28% (231)	4.65% (53)	7.64% (87)	14.05% (160)
	30-39 (N=1308)	15.14% (198)	22.71% (297)	8.79% (115)	23.55% (308)	14.14% (185)	3.59% (47)	2.68% (35)	16.13% (211)
	40-49 (N=909)	9.35% (85)	19.25% (175)	15.62% (142)	46.42% (422)	8.14% (74)	0.88% (8)	0.66% (5)	8.14% (74)
Race	White (N=1832)	22.71% (416)	23.36% (428)	11.85% (217)	22.11% (405)	14.52% (266)	1.80% (33)	3.00% (55)	14.74% (270)
	Black/AA (N=759)	12.65% (96)	38.87% (295)	1.58% (12)	21.21% (161)	16.60% (126)	8.43% (64)	4.61% (35)	10.41% (79)
	Hispanic (N=932)	13.41% (125)	28.76% (268)	3.86% (36)	22.64% (211)	15.45% (144)	4.40% (41)	5.47% (51)	12.77% (119)
	Other (N=188)	19.15% (36)	35.11% (66)	5.32% (10)	11.17% (21)	17.02% (32)	1.06% (2)	2.66% (5)	8.51% (16)
Education	High school level (N=461)	15.84% (73)	35.79% (165)	2.17% (10)	27.33% (126)	11.71% (54)	7.59% (35)	5.42% (25)	8.89% (41)
	High school degree (N=1015)	13.00% (132)	18.33% (286)	5.32% (54)	29.16% (296)	14.38% (146)	5.81% (59)	5.12% (52)	10.34% (105)
	College level (N=773)	18.76% (145)	30.92% (239)	6.34% (49)	18.50% (143)	18.24% (141)	3.49% (27)	4.53% (35)	13.45% (104)
	Undergraduate degree (N=1083)	22.35% (242)	26.32% (285)	10.25% (111)	16.44% (178)	16.16% (175)	1.57% (17)	2.31% (25)	15.97% (173)
	Higher education degree (N=358)	22.63% (81)	22.91% (82)	14.25% (51)	15.36% (55)	20.95% (76)	0.56% (2)	2.51% (9)	17.04% (61)
Marital status	Married to a man (N=1395)	13.48% (188)	17.63% (246)	23.08% (322)	29.39% (410)	11.04% (154)	1.22% (17)	3.23% (45)	13.48% (188)
	Not married, living with a man (N=571)	19.44% (111)	37.65% (215)	3.50% (20)	22.42% (128)	17.16% (98)	3.68% (21)	6.30% (36)	16.29% (93)
	Divorced (N=237)	11.81% (28)	28.27% (67)	7.59% (18)	42.19% (100)	13.08% (31)	1.27% (3)	2.53% (6)	14.77% (35)
	Separated (N=119)	9.24% (11)	26.89% (32)	2.52% (3)	36.13% (43)	10.92% (13)	5.04% (6)	1.68% (2)	15.13% (18)
	Never been married (N=1358)	24.45% (332)	43.74% (594)	0.88% (12)	8.39% (114)	19.96% (271)	6.85% (93)	5.30% (73)	10.90% (148)
Insurance status	Private, Med-Gap (N=2190)	22.42% (491)	27.12% (594)	10.55% (231)	18.54% (406)	14.75% (323)	2.28% (50)	2.47% (54)	14.75% (323)
	Medicaid, CHIP, state (N=820)	11.21% (92)	30.85% (253)	1.95% (16)	26.83% (220)	15.85% (130)	7.93% (65)	6.95% (57)	11.22% (92)
	Medicare, military, government (N=171)	17.54% (30)	22.22% (38)	9.36% (16)	27.49% (47)	15.20% (26)	4.09% (7)	5.26% (9)	13.45% (23)
	Single service, Indian, uninsured (N=509)	11.79% (60)	33.79% (172)	2.36% (12)	24.56% (125)	17.49% (89)	3.54% (18)	5.11% (26)	9.04% (46)

There were statistically significant differences (P < 0.001, for all categories) in the use of our selected methods of contraception (including OCPs, male condoms, partner's vasectomy, female sterilization, withdrawal, Depo-Provera, hormonal implant, and IUD) across different age groups. The use of different contraceptive methods significantly varied among race groups (OCPs: P < 0.001, male condoms: P < 0.001, partner’s vasectomy: P < 0.001, female sterilization: P = 0.005, Depo-Provera: P < 0.001, hormonal implant: P = 0.008, and IUD: P = 0.005). OCPs (P < 0.001), male condoms (P < 0.001), partner’s vasectomy (P < 0.001), female sterilization (P < 0.001), withdrawal (P = 0.001), hormonal implant (P = 0.002), and IUD (P < 0.001) use differed across education levels. The use of various contraceptives differed depending on marital status. OCPs, male condoms, partner's vasectomy, female sterilization, withdrawal, hormonal implant, and IUD use showed statistically significant differences in their usage across marital status (P < 0.001 for OCPs, male condoms, partner's vasectomy, and female sterilization; P = 0.002 for hormonal implant and IUD) Similarly, the usage of OCPs, male condoms, partner's vasectomy, female sterilization, Depo-Provera, hormonal implant, and IUD varied significantly with insurance status (P < 0.001 for all, except for IUD with P = 0.002) (Table 1).

All analyses presented in Table 1 were performed from raw NSFG data that were converted to frequencies, as shown in Table 2. Across the age groups, condoms were the most common form of contraception for age groups 15-19 and 20-29 (57.49% and 34.50%, respectively), whereas female sterilization was the most commonly reported for 30-39 and 40-49 groups (23.55% and 46.42%, respectively). All races, education levels aside from those with only high school degree completion (female sterilization: 29.16%), and insurance statuses reported condoms as the most frequently used form of contraception. Within the marital statuses, those who identified as married, divorced, or separated reported female sterilization to be most common (29.39%, 42.19%, and 36.13%, respectively), whereas those who were unmarried and living with a man used condoms most often (37.65%) (Table 2). Figure 1 exhibits a graphical representation of the frequencies represented in Table 2 across each social determinant of health and each contraceptive option.

## Discussion

These results demonstrate that contraceptive use among ages, races, education levels, marital statuses, and insurance statuses varies significantly and highlights patterns of contraceptive choice that clinicians may commonly encounter. As seen in Table 1, demographic parameters may exhibit a notable influence over contraceptive use, with nearly each of the eight methods holding statistically significant differences across all groups. Below, we highlight the most commonly reported contraceptive choices within the context of each social lens.

Regarding age, those in the age bracket of 15-19 usually opt for male condoms. This may be due to various reasons, such as the lack of familiarity with women’s health providers and the accessibility of male condoms. According to a study that focused on women aged 16 to 25, pregnancy prevention was the primary reason for using birth control [11]. However, the study found that these women were relying more on condoms, which happen to be one of the least effective methods, as opposed to long-acting reversible contraception (LARC) methods that are more effective [11]. It is important to be aware that women between the ages of 15 and 19 who rely on male condoms for birth control have a higher chance of contraceptive failure compared to women who are 40 years old and above [12]. Younger women often rely on recommendations from peers or family members for contraception instead of consulting healthcare providers, which is a critical factor to consider while educating and advising this group [11]. For those aged 20-29, male condoms were the most commonly used method, followed by OCPs. Of note, individuals less than the age of 25 are disproportionately affected by STIs [8]. Women aged 30-39 mostly opted for female sterilization and male condoms, while those aged 40-49 chose female sterilization as their preferred method. 

Regarding race, White women most commonly chose male condoms, followed very closely by OCPs and female sterilization. Black or African American women most commonly chose male condoms, followed by female sterilization and withdrawal. It is of note that, in Black or African American women, the percentage of male condom choice (38.87%) is nearly double that of female sterilization (21.21%). Hispanic women most commonly chose male condoms, followed by female sterilization and then withdrawal. It is interesting to note that, from the survey data, White women had a substantially higher rate of choosing OCPs than other races, and it raises the question of what barriers exist that have caused this, such as access to care, education around contraception, or other racial and ethnic contextual choices. One study found that racial and ethnic minority women, compared to White women, experience higher rates of contraceptive non-use, failure, unintended pregnancy, and lower use of LARC [13]. With racial and ethnic minority women facing higher rates of contraceptive failure and with male condom use having one of the highest failure rates (apart from withdrawal and periodic abstinence), it is notable that racial and ethnic minority women choose male condoms as their contraceptive method more often than other methods, compounding their already increased risk of contraceptive failure with a method that is known to carry greater risk and, ultimately, leaving them at even greater risk for unintended pregnancies [12,13]. One study found that when compared to White women, racial and ethnic minority women were statistically more likely to report certain features that were “extremely important” when selecting contraceptive methods, including being able to stop using the method at any time, using a method only with intercourse, the method not changing one’s menstrual periods, protection against STDs, having control over when and whether to use the method, and being able to become pregnant after discontinuation [14]. These findings imply that the higher rates of unintended pregnancies among racial and ethnic minority women may be attributed to differences in contraceptive features and preferences based on those features, rather than solely the socioeconomic, racial, or access barriers these groups may face [14]. This indicates the need to develop more acceptable contraceptive methods for these groups that not only are highly effective but also meet the aforementioned desired features [14]. 

Regarding education levels, those with either high school level education or a high school degree most commonly chose male condoms and female sterilization. Those with college level education most commonly chose male condoms. Those with an undergraduate degree most commonly chose male condoms, followed closely by OCPs. Individuals with higher education (a master’s, doctoral, or professional degree) showed no clear preference for any contraceptive method, but most commonly chose male condoms and OCPs. It is apparent that across all educational levels, male condoms are still one of the most commonly used contraceptive methods, likely due to multiple factors, including their ease of access, temporary nature, and presence as a well-known and accepted method. 

Regarding marital status, women who are married to a man most commonly elected for female sterilization, followed by a partner's vasectomy. Those who are not married but are living with a man most commonly opted for male condoms. Women who were divorced or separated most commonly chose female sterilization, followed by male condoms. Those who have never been married most commonly reported male condoms, followed by OCPs. These results delineate a clear preference for male condoms among those who have not been married compared to a preference for a more permanent or long-term means of contraception, including female sterilization or partner’s vasectomy in those who are married.

Regarding insurance status, those with private, Med-Gap, Medicaid, CHIP, state, single service, or Indian insurance or were uninsured most commonly elected male condoms as their primary form of contraception. Those with Medicare, military, or government insurance, on the other hand, most commonly chose female sterilization. Female sterilization was the second most common choice by individuals with Medicaid, CHIP, state, single service, and Indian insurance and uninsured individuals. The OCP was the second most common choice by individuals with private or Med-Gap insurance, and the male condom was the second most common choice by individuals with Medicare, military, or government insurance. Failure rates for OCPs, condoms, withdrawal, and periodic abstinence are substantially higher for women in the lowest quintile socioeconomic level of the population than women from the wealthiest [12]. It has been shown that contraceptive failure disproportionately affects both the youngest and poorest women, which puts these women at risk for unintended pregnancy and therefore at risk for poor maternal and birth outcomes [12]. Women who are young and economically disadvantaged often struggle to access proper care for unintended pregnancies, putting both mother and baby at risk [12].

It is clear through multiple studies that socioeconomic status and race overlap and may compound barriers to care that patients of minority racial and ethnic backgrounds may face. For example, one study found that compared to White women with commercial insurance, Black and Hispanic women with Medicaid had higher odds of receiving LARC, suggesting implicit biases that may influence contraception counseling [15].

In addition, multiple studies have been done to identify changes in patterns of contraceptive use when financial barriers are removed. In one study that sought to reduce unintended pregnancies by removing barriers to care including cost, education, and access, researchers found that 75% of participants chose LARC as a contraceptive method [16]. In a follow-up study, LARC methods were found to reduce the percentage of repeat abortions and substantially lower teen pregnancy, birth, and abortion rates compared to national rates [16]. In a study aimed at reducing the impact of socioeconomic disadvantage by providing clients with free access to a new birth control method, researchers discovered that individuals facing such disadvantage were less likely to choose any IUD [17]. In addition, those who identified as Black had a higher likelihood of choosing the three-month injectable, while those who identified as multiracial had a greater chance of selecting the vaginal ring [17]. This study demonstrates that eliminating financial barriers to care does not eliminate the socioeconomic contexts that occur as a part of a patient’s lived experiences and that may influence contraceptive choice [17]. 

Of note, it has been found that LARC users report lower rates of consistent condom use, but with no change when compared to preinitiation of LARC in those individuals [18]. In addition, LARC users were more likely to acquire an STI in the 12 months after initiation [18]. This highlights the continuous need for education surrounding and promoting STI-preventing contraception, such as male condoms, even when long-term contraception is in place. 

Disparities are interesting in how they overlap. For example, as previously mentioned, although ethnic and racial minorities are more likely to choose condoms and more likely to choose methods that help prevent STDs [14], these groups are still at a much higher risk for STIs [19]. A review of national case report data shows the large disparities that exist in STI prevalence among races, finding that rates of congenital syphilis, gonorrhea, chlamydia, HIV/AIDS, and primary/secondary syphilis were 19.9, 17.8, 8.2, 8.1, and 5.4 times higher, respectively, for the Black and African American race when compared to the White race [19]. 

While some forms of birth control are more effective than others, the American College of Obstetrics and Gynecology (ACOG) does not consistently recommend one form over the other, as the selection of birth control should be individualized for each patient [20]. Based on our analysis, when selecting a birth control method, we encourage providers to have a thorough discussion with their patients on the advantages and disadvantages of each method, so that they can decide on the best form for the patient’s beliefs, interests, and medical needs. 

Limitations and strengths

Our study has potential limitations. Our project is not aimed at establishing cause-and-effect relationships between data points. Rather, our objective was to identify patterns within the data and draw inferences based on existing literature to account for their existence. It is important to recognize that there are numerous factors that contribute to obstacles in accessing care, and tackling only one may not be sufficient to overcome all of the challenges or institutionalized barriers that affect a particular group. Inherent strengths within this study design include the large generalizable sample size, statistical weighting within the survey data, and the utilization of three consecutive years of the most recently collected survey data.

Further research is necessitated to define these patterns more clearly and further investigate the multifaceted causal relationships that may exist among these patterns. In addition, these patterns are based on data retrieved from 2017 to 2019. It would be interesting to explore how patterns may have shifted since the Dobbs vs. Jackson Women's Health Organization case in June 2022. This decision overturned the Supreme Court's previous ruling in Roe vs. Wade, which protected a woman's right to end a pregnancy in its early stages [21]. Further research should be conducted in this area to analyze the effects of this decision.

## Conclusions

This analysis highlights gaps that are present in female reproductive autonomy through the statistical differences in contraceptive methods across various demographics and warrants continued focus on the role that social determinants of health play in the prevention of unintended pregnancies and STIs. Healthcare providers should be cognizant of the multifactorial barriers that are affecting their patients’ access and acceptance of contraceptive methods and utilize a culturally competent approach in educating and counseling patients on contraception. Healthcare providers should be diligent in educating patients on the importance of preventing STIs and should delineate the difference between contraception that serves to prevent unintended pregnancies and those that serve to prevent STIs.

This study also highlights the disparities that exist among multiple demographics in their access to, understanding of, and preference for contraceptive methods. Unplanned pregnancies and the spread of STIs remain a pressing public health issue, both domestically and internationally. In order to promote fairness and equality in healthcare, it is essential to increase education on these topics both within and beyond medical settings. This effort aims to provide patients with equitable access to healthcare and attempt to address health disparities that are prevalent in multiple different sectors.
